# Intraday repeatability of macular layers measurements in glaucomatous and non-glaucomatous patients using spectral-domain optical coherence tomography

**DOI:** 10.1007/s00417-024-06498-7

**Published:** 2024-05-06

**Authors:** Vadim Makhmutov, Werner Adler, Philomena A. Wawer Matos, Adam Kopecky, Jan Nemcansky, Alexander C. Rokohl, Ludwig M. Heindl

**Affiliations:** 1 Department of Ophthalmology, Faculty of Medicine and University Hospital of Cologne, University of Cologne, Cologne, Germany; 2 Department of Medical Informatics, Biometry, and Epidemiology, Friedrich-Alexander, University Erlangen-Nuremberg, Erlangen, Germany; 3 Clinic of Ophthalmology, University Hospital Ostrava, Ostrava, Czech Republic; 4 Department of Craniofacial Surgery, University of Ostrava – Faculty of Medicine, Ostrava, Czech Republic

**Keywords:** Spectral-domain optical coherence tomography, Glaucoma progression analysis, Posterior pole analysis

## Abstract

**Purpose:**

To assess the intraday repeatability of macular architecture measurements in glaucomatous and non-glaucomatous patients using spectral-domain optical coherence tomography (SD-OCT) and to evaluate the independence from intraindividual intraocular pressure (IOP) fluctuations.

**Methods:**

In this single-center, time-point comparison study, 88 eyes with glaucoma, 53 eyes with ocular hypertension (OHT), and 253 healthy eyes underwent two standardized SD-OCT and intraocular pressure (IOP) measurements on the same day with a 5-h time gap. Bland–Altman plots, intraclass correlation coefficients (ICC), and random-effects model were used to analyze repeatability of entire retinal thickness, retinal nerve fiber layer, ganglion cell layer, inner plexiform layer, and inner nuclear layer measurements.

**Results:**

Intraday measurements were highly reproducible in all 3 groups. ICC were greater than 0.90, respectively. The pairwise comparisons of morphometric parameters showed a statistically significant difference (P < 0.001, respectively) between groups (glaucoma vs. control, glaucoma vs. OHT) and a significant influence of time points. No correlation was found between IOP fluctuations and morphometric parameters (*P* > 0.05, respectively), except for a weak positive correlation with GCL (rho = 0.109, *P* = 0.031).

**Conclusions:**

The evaluation of macular morphometric parameters of SD-OCT showed a high intraday repeatability and an excellent degree of agreement in glaucoma, ocular hypertension, and healthy groups. The fixed effects of time points were statistically significant. Except for a weak positive correlation of ganglion cell layer, variability did not appear to be affected by intraday IOP changes. Additional research is required to fully understand the impact of IOP fluctuations on macular morphometric parameters, considering the small observed IOP changes.


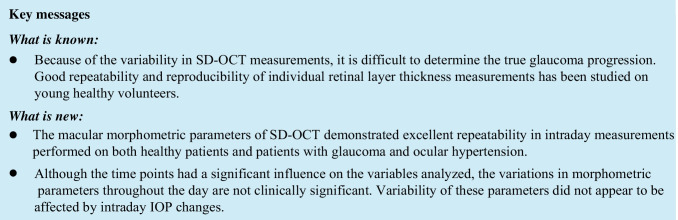


## Introduction

Glaucoma is a heterogeneous group of optic nerve diseases and leads to characteristic morphological changes of the optic nerve (increasing optic disc excavation), loss of retinal ganglion cells, and thinning of the retinal nerve fiber layer [1–3]. The specific visual field changes are also the main features of this chronic progressive optic neuropathy. If left untreated, glaucoma leads to severe visual acuity loss even to permanent blindness [1, 4, 5].

Thanks to modern technological advances in the last decades, spectral-domain optical coherence tomography (SD-OCT) has become a significant and established examination technique in ophthalmology and the diagnosis of glaucoma in particular [6–8]. SD-OCT measurements in glaucoma are based on Bruch's membrane opening and include key quantitative measurements such as an analysis of BMO-minimal rim width, circumpapillary retinal nerve fiber layer thickness, and macular architecture in the posterior pole [7, 9]. Compared with confocal scanning laser ophthalmoscopy (cSLO), which was the gold standard until recently, SD-OCT is a more reliable method for morphometric examination of the optic disc [6, 10–16].

In diagnosing glaucoma with SD-OCT, the most significant and frequently used parameter is the assessment of the circumpapillary retinal nerve fiber layer thickness of the optic nerve head [15, 17–20]. However, assessment of macular architecture (especially thickness of retinal nerve fiber layer, ganglion cell layer, and inner plexiform layer) can serve as an important additional diagnostic tool in the early diagnosis of glaucoma [17, 21–24]. With more advanced and highly reproducible segmentation algorithms of all retinal layers in the macular region, this type of analysis is of interest for clinical use in the detection of glaucomatous lesions [9, 17, 25–27].

In addition, with an increasing number of follow-up SD-OCT examinations in glaucoma thanks to its ubiquitous use, the logical question arises to what extent intraindividual measurement variability may influence the results of comparative progression analyses [2, 6, 17]. Repeated precise measurements are essential for a correct glaucoma progression analysis of intraindividual disease dynamics [2, 7].

There is a hypothesis that variations in the anatomical structures of the optic nerve are possible due to intraday variations in intraocular pressure (IOP) and blood flow of the optic nerve [19, 28]. Such fluctuations in anatomical structures may also be characteristic of the retina in the macula region and hinder the accurate detection of glaucoma progression depending on the time of day when SD-OCT coherence tomography is performed [17].

Knowing the presence and quantitative values of intraday variations of macular architecture, the true glaucomatous progression and measurement variability of morphometric parameters can be distinguished. However, there is a lack of confirmed data on this to date. Therefore, the purposes of this study are to assess the intraday repeatability of macular architecture measurements in glaucomatous and non-glaucomatous patients using spectral-domain optical coherence tomography and to evaluate the independence from intraindividual intraocular pressure fluctuations.

## Materials and methods

The present single-center, time-point comparison study was conducted between February 2020 and June 2021 in a German primary ophthalmology center. All procedures performed in this study were following the ethical standards of the institutional and/or national research committee and with the 1964 Helsinki declaration and its later amendments or comparable ethical standards. Approval for the study was obtained from the Ethics Commission of Cologne University’s Faculty of Medicine (19–1663). Written informed consent was obtained from all participants.

In this study, SD-OCT and Goldmann applanation tonometry were performed at two different time points with a time interval of 5 h on the same day (morning and afternoon). The following inclusion criteria were considered: the ability to give consent or consent by the legal representative, sufficient fluency in the German language to understand the contents of this study, and good cooperation of the patient. The following patients were excluded from this study: underage patients, pregnant patients, patients with a current or past history of systemic diseases with ocular involvement, retinal vascular diseases, visual field defects of other origins than glaucoma, high myopia greater than 6.00 dpt, pathological changes of the macular region or the optic nerve, severe opacities of the refractive media. Patients were classified into the three diagnostic groups according to the 5th edition of the European Glaucoma Society (EGS) guidelines: glaucoma group, ocular hypertension (OHT) group, and healthy patients without glaucoma (control group) [29].

Spectral Domain optical coherence tomography was performed according to the general guidelines using the Glaucoma program (scan size in the macular region 25 × 30°, central position of the pattern, number of scans of the region 61, distance between the regions 120 µm). The SD-OCT Spectralis® Glaucoma Module Premium Edition (Heidelberg Engineering GmbH, Heidelberg, Germany) was used with the device operating software version 6.13. The focus of the study was posterior pole analysis (8 × 8 grid with 64 squares pro layer) and measurement of the following morphometric parameters: entire retinal thickness (RT), the thickness of retinal nerve fiber layer (RNFL), ganglion cell layer (GCL), inner plexiform layer (IPL), and inner nuclear layer (INL). The minimal accepted quality of SD-OCT scans was 15 dB.

### Statistical analyses

Patient data were pseudonymized, analyzed, and graphed using IBM SPSS Statistics version 27.0 (IBM Corp. Armonk, NY, USA). Dependencies between groups, time points and macular morphometric parameters were examined using random-effects models. Type C intraclass correlation coefficients were calculated for quantifying the agreement of morphometric parameters. The Bland–Altman graph was used for graphical assessment. Spearman's rank correlation analyses were used to test correlations between morphometric parameters and IOP. The threshold for statistical significance was *P* < 0.05.

## Results

### Demographics

In this study, 221 patients (442 eyes) were enrolled. Both eyes of the participants were included in the study. The insufficient image quality of SD-OCT scans (i.e., the quality of scans less than 15 dB) led to exclusion of 18 eyes from the study. In addition, 15 patients (30 eyes) dropped out since they did not participate in the second examination. Subsequently, the 394 eyes (204 patients) with a mean age of 58.14 ± 12.57 years were included in the statistical analyses (Table 1).

**Table 1:** Demographic and baseline data of the subjects

Groups	Glaucoma group (N = 88)	OHT group (N = 53)	Control group (N = 253)
Gender, *n* (%)			
men	39 (44.3%)	16 (30.2%)	91 (36.0%)
women	49 (55.7%)	37 (69.8%)	162 (64.0%)
Eye, *n* (%)			
right	43 (48.9%)	28 (52.8%)	128 (50.6%)
left	45 (51.1%)	25 (47.2%)	125 (49.4%)
Age, *y*			
Mean ± SD	64.39 ± 8.88	58.51 ± 11.73	55.89 ± 13.12
Median	65.50	59.00	59.00
Lens status, *n* (%)			
clear lens	43 (48.9%)	37 (69.8%)	189 (74.7%)
cataract	35 (39.7%)	15 (28.3%)	51 (20.2%)
pseudophakia	10 (11.4%)	1 (1.9%)	13 (5.1%)
BCVA (LogMAR)			
Mean ± SD	0.05 ± 0.09	0.02 ± 0.05	0.01 ± 0.05
Spherical equivalent, D			
Mean ± SD	0.24 ± 1.39	0.17 ± 1.78	0.24 ± 1.69
Range	-4.37 – 2.50	-5.50 – 4.75	-4.50 – 4.38
IOP, mmHg, Mean ± SD			
first examination	16.06 ± 3.90	21.92 ± 2.82	15.64 ± 2.85
second examination	15.31 ± 3.74	20.32 ± 3.14	14.73 ± 2.94
maximal IOP in medical history	26.36 ± 5.85	24.64 ± 2.26	17.00 ± 2.62
Mean Deviation, dB			
Mean ± SD	-3.30 ± 4.05	-1.47 ± 2.11	-1.33 ± 1.95

*OHT* ocular hypertension, *dB* decibel, *IOP* intraocular pressure, *D* diopters, *SD* standard deviation

The first SD-OCT examinations were performed in the morning between 7:10 a.m. and 12:43 p.m. (9:40 ± 1:12), whereas the second SD-OCT examinations were performed between 1:04 p.m. and 5:15 p.m. (3:17 ± 0:51). The difference between the first and second examination averaged 5 h 36 min (336 ± 60 min). The mean value of the focus scan was 0.46 ± 1.64 diopters in the first SD-OCT examination and 0.45 ± 1.63 diopters in the second SD-OCT examination. Table 2 provides mean of macular morphometric parameters and their absolute difference in two time points.

**Table 2:** Mean of morphometric parameters and their absolute difference in two time points

Retinal layers	Glaucoma group			OHT group			Control group		
	First examination Mean ± SD	Second examination Mean ± SD	Absolute difference Mean ± SD	First examination Mean ± SD	Second examination Mean ± SD	Absolute difference Mean ± SD	First examination Mean ± SD	Second examination Mean ± SD	Absolute difference Mean ± SD
RT, µm	282.19 ± 14.51	281.62 ± 14.83	1.80 ± 1.81	293.70 ± 14.92	292.88 ± 14.59	1.52 ± 1.27	292.63 ± 11.51	291.99 ± 11.35	1.37 ± 1.16
RNFL, µm	34.61 ± 7.72	34.57 ± 7.82	0.83 ± 0.95	41.39 ± 4.99	41.60 ± 4.96	0.83 ± 1.09	41.55 ± 4.72	41.57 ± 4.62	0.63 ± 0.68
GCL, µm	28.73 ± 3.92	28.68 ± 3.85	0.43 ± 0.44	32.61 ± 2.30	32.43 ± 2.26	0.40 ± 0.37	32.62 ± 2.50	32.51 ± 2.46	0.37 ± 0.36
IPL, µm	24.90 ± 2.34	24.82 ± 2.32	0.38 ± 0.39	26.90 ± 1.74	26.66 ± 1.63	0.39 ± 0.46	27.02 ± 1.99	26.81 ± 1.94	0.36 ± 0.50
INL, µm	31.69 ± 2.58	31.74 ± 2.60	0.53 ± 0.62	31.03 ± 1.72	31.02 ± 1.72	0.45 ± 0.34	30.89 ± 1.78	30.99 ± 1.77	0.40 ± 0.36

*OHT* ocular hypertension, *RT* entire retinal thickness, *RNFL* retinal nerve fiber layer, *GCL* ganglion cell layer, *IPL* inner plexiform layer, *INL* inner nuclear layer, *SD* standard deviation

### Bland–Altman plot

**Fig. 1:**
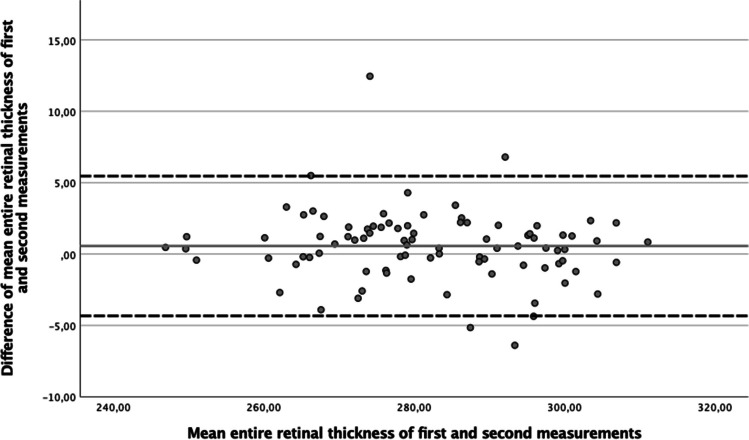
Bland-Altmann plot showing the intraday repeatability of two measurements (*n* = 88) of the entire retinal thickness in the glaucoma group

There was a good agreement of the SD-OCT measurements in all 3 groups and the macular morphometric measurements were repeatable between examinations at two different time points during the same day. The mean of the difference of morphometric parameters in two time points was in the glaucoma group for RT = 0.57 ± 2.50 µm, RNFL = 0.05 ± 1.26 µm, GCL = 0.05 ± 1.62 µm, IPL = 0.08 ± 0.54 µm, and INL = -0.05 ± 0.81 µm, in the OHT group for RT = 0.83 ± 1.80 µm, RNFL = -0.20 ± 1.36 µm, GCL = 0.17 ± 0.51 µm, IPL = 0.24 ± 0.56 µm, and INL = 0.01 ± 0.57 µm, in the control group for RT = 0.64 ± 1.68 µm, RNFL = -0.01 ± 0.93 µm, GCL = 0.11 ± 0.51 µm, IPL = 0.21 ± 0.58 µm, and INL = -0.10 ± 0.53 µm and mostly distributed within standard deviations (± 1.96 SD). The mean values were in a very narrow range. There was no relationship of differences and decreasing or increasing means of morphometric parameters (Fig. 1).

### Intraclass correlation coefficients (ICC)

The 95% confidence intervals of ICC in the glaucoma group were from 0.988 to 0.995 for RT (*P* < 0.001), from 0.990 to 0.996 for RNFL (*P* < 0.001), from 0.990 to 0.996 for GCL (*P* < 0.001), from 0.979 to 0.991 for IPL (*P* < 0.001), and from 0.962 to 0.984 for INL (*P* < 0.001), in the OHT group from 0.990 to 0.998 for RT (*P* < 0.001), from 0.967 to 0.989 for RNFL (*P* < 0.001), from 0.974 to 0.992 for GCL (*P* < 0.001), from 0.933 to 0.983 for IPL (*P* < 0.001) and from 0.952 to 0.984 for INL (*P* < 0.001), in the control group from 0.990 to 0.996 for RT (*P* < 0.001), from 0.987 to 0.992 for RNFL (*P* < 0.001), from 0.985 to 0.992 for GCL (*P* < 0.001), from 0.962 to 0.983 for IPL (*P* < 0.001), from 0.970 to 0.982 for INL (*P* < 0.001) (Table 3).

**Table 3:** Type C intraclass correlation coefficients of the macular morphometric parameters between two intraday measurements

Retinal layers	Glaucoma group	OHT group	Control group
Entire retinal thickness	0.992 (0.988–0.995)	0.996 (0.990–0.998)	0.994 (0.990–0.996)
Retinal nerve fiber layer	0.993 (0.990–0.996)	0.981 (0.967–0.989)	0.990 (0.987–0.992)
Ganglion cell layer	0.994 (0.990–0.996)	0.986 (0.974–0.992)	0.989 (0.985–0.992)
Inner plexiform layer	0.986 (0.979–0.991)	0.967 (0.933–0.983)	0.975 (0.962–0.983)
Inner nuclear layer	0.975 (0.962–0.984)	0.972 (0.952–0.984)	0.976 (0.970–0.982)

The values of these parameters reflect mean and 95% confidence intervals. OHT—ocular hypertension

### Random-effects model

The fixed effect of the group variable was statistically significant (*P* < 0.001) in three groups. The pairwise comparisons of morphometric parameters (RT, RNFL, GCL, IPL) showed a statistically significant difference between the glaucoma group and the OHT group (*P* < 0.001) and between the glaucoma group and the control group (*P* < 0.001) and no statistically significant difference between the OHT group and the control group. The fixed effects for time points (RT: η2 = 0.1055, *P* < 0.001; GCL: η2 = 0.0374, *P* < 0.001; IPL: η2 = 0.0944, *P* < 0.001; INL: η2 = 0.0141, *P* = 0.018) were statistically significant, except in the case of RNFL (η2 = 0.0006, *P* = 0.635).

### The Spearman's rank coefficient of correlation

The mean IOP was 16.58 ± 3.76 mmHg (*min* = 8 mmHg, *max* = 29 mmHg) at the first examination and 15.61 ± 3.67 mmHg (*min* = 6 mmHg, *max* = 28 mmHg) at the second examination. The intraindividual absolute mean difference of IOP between the two measurements at two different time points was 1.82 ± 1.66 mmHg. The intraindividual absolute mean variability of morphometric parameters among patients in all three groups was 1.49 ± 1.35 µm for RT, 0.70 ± 0.81 µm for RNFL, 0.39 ± 0.38 µm for GCL, 0.37 ± 0.47 µm for IPL and 0.44 ± 0.43 µm for INL. No statistically significant correlation was found between absolute intraindividual variability of IOP and absolute difference of RT (rho = 0.088, *P* = 0.082), RNFL (rho = 0.087, *P* = 0.085), IPL (rho = -0.035, *P* = 0.488) and INL (rho = 0.040, *P* = 0.435). A statistically significant weak positive correlation was found between the mean absolute difference of IOP and the mean absolute difference of GCL (rho = 0.109, *P* = 0.031).

## Discussion

Timely detection of the progression of optical neuropathy is an important aspect of glaucoma management since early recognition of changes in optic nerve morphology and macular architecture can help clinicians to adjust patient treatment and prevent permanent vision loss [30]. Accurate and reliable SD-OCT measurements are crucial for a correct analysis of the glaucoma progression and dynamics of the disease [2, 7]. Difficulties in right diagnosing of the glaucoma progression include not only the complexity of distinguishing true changes associated with the disease from normal aging processes of the retina but also the variability of SD-OCT measurements themselves [17, 31].

In this study, we could show that the macular morphometric parameters (RT, RNFL, GCL, IPL, INL) have a high degree of agreement between the measurements in the morning and the afternoon. According to Bland–Altman plots and intraclass correlation coefficients, it can be concluded that the variations of the morphometric parameters during the day are not clinically significant and that the measurement results in all three groups (glaucoma, ocular hypertension, and healthy groups) speak for the overall excellent repeatability.

Ctori et al. also reported good repeatability and reproducibility of the individual measurements of the retinal layer thickness in young healthy volunteers. In this study, two investigators independently performed two SD-OCT examinations for each participant in a single visit. In the literature, there are also works devoted to the assessment of the variability of morphometric parameters of the optic nerve head. Enders et al. reported high intraday repeatability after assessing the variability of global BMO-MRW, global RNFL thickness, and global BMO-MRA at two different time points with a time gap of more than 5 h [19]. 

On the other hand, the analysis using random-effects model indicated a statistically significant influence of time points on a measurement of macular architecture. This variability between intraday measurements could be explained by the inability of performing an absolutely identical SD-OCT examination in practice, due to the patient cooperation during the examination and the skills of the staff performing tomography. The fluctuating quality of SD-OCT images can play a crucial role in the variation in the thickness of the retina and its layers since the correct determination of the boundaries of these layers is done automatically by the software of the OCT device. The higher the quality of the images, the more correctly the segmentation algorithms of the software work and, consequently, the more accurately thickness of retinal layers is detected. Each performed examination should also be verified by a physician and manual corrections should be made to the automatic segmentation of the retinal layers, if necessary [7, 32]. As has already been confirmed, manual segmentation correction increases the diagnostic power of another SD-OCT morphometric parameter such as circumpapillary RNFL to detect glaucomatous changes of the retina [9, 33, 34].

The absolute mean variability of the RT, RNFL, IPL, and INL did not correlate in our study with the absolute IOP variations on the two SD-OCT examinations performed. The Spearman correlation of the mean absolute difference of the GCL and the mean absolute difference of the IOP has a value of rho = 0.109 (*P* = 0.031) and can be described as weak positive correlation, because it is in the range of 0.1 to 0.3 [35]. However, it cannot be claimed why there is a weak positive correlation only with ganglion cell layer. Possibly, this layer of the retina is more susceptible to IOP variations than the other retinal layers or the entire retina itself. Further studies are necessary to gain a comprehensive understanding of the potential impact of IOP changes on macular morphometric parameters, due to the small detected intraday IOP fluctuations. Enders et al. found no significant correlation between intraindividual variability in morphometric parameters of the optic nerve head (global BMO-MRW, global RNFL thickness, global BMO-MRA) and intraindividual IOP changes in two examinations performed on the same day [19].

Limitations of this work include the relatively small number of patients in the glaucoma group and the OHT group. In this study, only the inner retinal layers were examined because of the already proven changes that occur in them by the progression of glaucoma [23, 24]. To obtain a complete picture of the possible intraindividual variations of macular morphometric parameters during the day, it would be useful to study all retinal layers. The good documentation of the morphometric parameters of the retina thanks to high-resolution SD-OCT is an advantage of this work. The variation of retinal vascular characteristics of the macular region and its possible influence on intraindividual variation in macular morphometric measurements should be investigated with the help of optical coherence tomography angiography (OCT-A) in future studies.

In summary, the data of this study showed that the macular morphometric parameters of SD-OCT demonstrated excellent repeatability in two measurements performed in the morning and afternoon (time interval of 5 h 36 min). According to intraclass correlation coefficients, the degree of agreement of each morphometric parameter in three studied groups in two time points was very good. The time points had a significant influence on studied variables. The fluctuation of thickness of the RT RNFL, IPL, and INL does not seem to be affected by the moderate variations of IOP. There was a weak positive correlation between the mean absolute difference of IOP and the mean absolute difference of GCL.

## Data Availability

The data that support the findings of this study are available on request from the corresponding author. The data are not publicly available due to privacy or ethical restrictions.
